# A Glutamate Scavenging Protocol Combined with Deanna Protocol in SOD1-G93A Mouse Model of ALS

**DOI:** 10.3390/nu15081821

**Published:** 2023-04-10

**Authors:** Christopher Q. Rogers, Melissa Ramirez, Carol S. Landon, Janine M. DeBlasi, Andrew P. Koutnik, Csilla Ari, Dominic P. D’Agostino

**Affiliations:** 1 Department of Molecular Pharmacology and Physiology, Laboratory of Metabolic Medicine, Morsani College of Medicine, University of South Florida, Tampa, FL 33612, USA; mramirez54655@gmail.com (M.R.); ddagosti@usf.edu (D.P.D.); 2 Human Healthspan, Resilience and Performance, Florida Institute for Human and Machine Cognition, 40 S Alcaniz St, Pensacola, FL 32502, USA; 3 Department of Psychology, University of South Florida, Tampa, FL 33612, USA; 4 Ketone Technologies LLC, 12608 Forest Hills Dr, Tampa, FL 33612, USA

**Keywords:** blood glutamate scavengers, oxaloacetic acid, medium chain triglycerides, Deanna Protocol, CoQ10, amyotrophic lateral sclerosis, motor neuron disease, neurodegeneration

## Abstract

Amyotrophic lateral sclerosis (ALS) is a progressive disease of neuronal degeneration in the motor cortex, brainstem, and spinal cord, resulting in impaired motor function and premature demise as a result of insufficient respiratory drive. ALS is associated with dysfunctions in neurons, neuroglia, muscle cells, energy metabolism, and glutamate balance. Currently, there is not a widely accepted, effective treatment for this condition. Prior work from our lab has demonstrated the efficacy of supplemental nutrition with the Deanna Protocol (DP). In the present study, we tested the effects of three different treatments in a mouse model of ALS. These treatments were the DP alone, a glutamate scavenging protocol (GSP) alone, and a combination of the two treatments. Outcome measures included body weight, food intake, behavioral assessments, neurological score, and lifespan. Compared to the control group, DP had a significantly slower decline in neurological score, strength, endurance, and coordination, with a trend toward increased lifespan despite a greater loss of weight. GSP had a significantly slower decline in neurological score, strength, endurance, and coordination, with a trend toward increased lifespan. DP+GSP had a significantly slower decline in neurological score with a trend toward increased lifespan, despite a greater loss of weight. While each of the treatment groups fared better than the control group, the combination of the DP+GSP was not better than either of the individual treatments. We conclude that the beneficial effects of the DP and the GSP in this ALS mouse model are distinct, and appear to offer no additional benefit when combined.

## 1. Introduction

Amyotrophic lateral sclerosis (ALS) is the most common motor neuron disease in adults, with a yearly incidence of two cases per 100,000 and a prevalence of six cases per 100,000. The average age of onset is 58–60 years, with an average survival of between 3 and 4 years [1]. It is estimated that in the United States, there are currently 30,000 cases. Signs and symptoms of ALS include difficulty walking, speaking, and swallowing, muscle fasciculations and weakness, anorexia and cachexia, hyperreflexia, and progressive paralysis. Critical to the progression of this disease, the majority of ALS patients experience cachexia, decreased strength, and loss of appetite, often associated with dysphagia [2].

About 90% of ALS cases have no known cause and are termed *sporadic* ALS, and are presumably the effect of unidentified environmental exposures [3], infection [4,5], or perhaps unidentified *de novo* mutations. In the rest of the cases, there is a history of disease in the family, and they are thus referred to as *familial* ALS. Of these cases, about 20% have been mapped to the SOD1 gene located on chromosome 21, where over 200 individual mutations have been identified [6]. This gene codes for superoxide dismutase 1 (SOD1), which is a cytosolic Cu/Zn-dependent enzyme and a critical component of the humoral anti-oxidative system.

The most widely used animal model for researching ALS is the SOD1-G93A mouse model, which contains a substitution mutation at amino acid 93, specifically glycine replaced with alanine, homologous to ALS in humans [7]. Although rare, it is thought that the mutation causes a toxic gain of function in ALS patients, leading to neurodegeneration [8]. Indeed, as SOD-1 has been shown to inhabit several cellular compartments, including the cytosol, the nucleus, and the mitochondria, its function may depend on its subcellular environment [9]. While it is unclear whether the effects in mice are related to abnormal aggregation of the misfolded protein and/or changes in oxidative damage [10], this mutation does confer a phenotype with remarkable fidelity to the pathology seen in humans, which explains its widespread use in research.

The pathophysiology of ALS in humans is linked to impaired cell signaling and axonal transport, protein aggregates, and mitochondrial abnormalities. Another prominent feature of ALS is glutamate excitotoxicity, which is pathophysiologically linked to neuronal dysfunction and cell death. While there are currently four drugs approved by the U.S. FDA to treat ALS (Radicava, Rilutek, Tiglutik, and Nuedexta), their efficacy is equivocal. The most widely used of these is Rilutek, which has been shown to extend the median time to tracheostomy or death by 2 or 3 months [11]. It appears to slow the progression of the disease by blocking NMDA receptors and reducing voltage-dependent sodium channels on glutamatergic nerve terminals, thus reducing the level of CNS glutamate [12], which is typically elevated in ALS patients [13].

To combat the effects of elevated glutamate in the CNS, blood glutamate scavenging treatments have been developed. By removing peripheral blood glutamate, a sink effect can be created, whereby CNS glutamate is transported unidirectionally into the periphery by facilitated diffusion through brain endothelial cells [14]. This effect is achieved through the activity of the endogenous enzyme serum glutamic oxaloacetic transaminase ((sGOT or GOT), also known as aspartate transaminase or aspartate aminotransferase (AST)), which catalyzes the transfer of the amino group from glutamate to oxaloacetate in exchange for a carbonyl group. The products of this reversible reaction are alpha-ketoglutarate and aspartate:

Glutamate + Oxaloacetate ↔ Alpha-Ketoglutarate + Aspartate

Oxaloacetate (Ox) is an anaplerotic molecule and provides acetyl CoA with an entry point into the TCA cycle. Treatment with GOT and/or Ox has been shown to increase the removal of glutamate from the blood and the CNS, thereby reducing excitotoxicity and improving neuronal health in numerous studies [15,16,17], including a rat model of ALS [18]. While either GOT or Ox alone has been shown to produce favorable results on CNS glutamate toxicity, the combination of GOT and Ox is even more effective and also allows for reduced levels of Ox and reduced acid load [16].

It has been demonstrated previously that Ox alone can provide life extension in a *C. elegans* model [19]. This was dependent on the FOXO–AMPK pathway independent of CNS glutamate scavenging and illustrates another powerful effect of Ox. Ox is an important intermediate in gluconeogenesis, fatty acid synthesis, and amino acid synthesis under normal conditions.

A novel metabolic therapy known as the Deanna Protocol (DP) was designed to support mitochondrial function and provide alternate mechanisms for energy production for patients with ALS. It consists of four commercially available dietary supplements (arginine:alpha-ketoglutarate, MCT oil, β-phenyl-GABA, and coenzyme Q_10_). In a previous study, we found that the DP improved motor function, delayed neurological deficits, and extended survival in SOD1-G93A mice [20]. In addition, the DP produced a change in the global metabolic profile of SOD1-G93A mice that supports the role of the DP for enhanced mitochondrial energy metabolism, neuronal homeostasis, and prolonged time to paralysis of the transgenic C. elegans TDP-43 ALS disease model [21]. Furthermore, research using human SOD1 patient-derived iPSCs demonstrated that the DP ameliorated glutamate-induced axonal varicosities by over 70% [22]. The data from three different model systems, including ALS patient-derived cells, are consistent with the anecdotal reports over the last decade of the DP alleviating symptomatic disease progression in ALS patients (V. Tedone, personal communication 2015).

In this study, we present new findings on the effect of three different treatments on the SOD1-G93A ALS mouse model: the DP alone, a blood glutamate scavenging treatment alone, and a combination of the two. One goal of this project was to repeat the DP treatment to further investigate its potential as a therapeutic approach. Further, we hypothesized that the glutamate scavenging protocol would lead to improvements in the ALS mice, and since the mechanism is distinct from the DP, we predicted the combination of approaches would provide additional benefit.

## 2. Materials and Methods

### 2.1. Ethical Declaration

All animal procedures were performed in compliance with the NIH Guide for the Care and Use of Laboratory Animals. Animals were used with permission of, and under the guidance of, the USF Institutional Animal Care and Use Committee (IACUC # IS00001563). The present study closely adhered to guidelines for preclinical research in ALS/MND [23].

### 2.2. Pharmacokinetic Experiment

**Figure 1:**
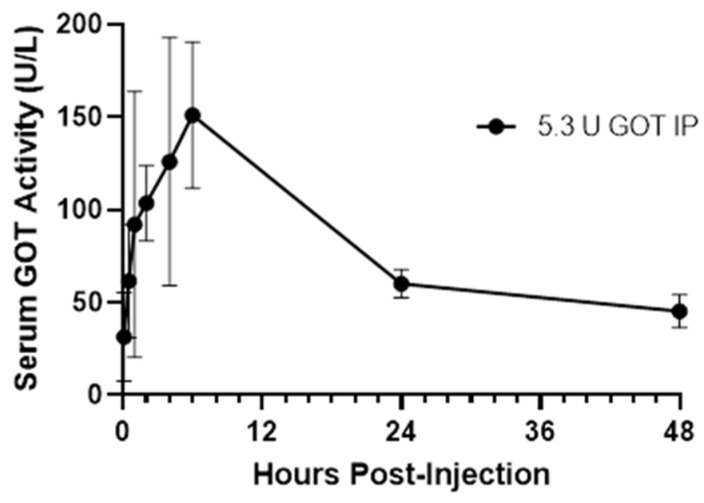
Time course of serum GOT levels. Serum was taken at various time intervals following intraperitoneal injections of 5.3 units glutamic oxaloacetate transaminase (GOT) into B6SJLF1/J mice (n = 3 or 4) (Jax Labs stock # 100012). After reaching an apparent maximum at 6 h, elimination occurred with a decay rate of 102.5 h with a T1/2 of 18 h. Data points indicate mean +/− SEM.

In order to ensure that the glutamic oxaloacetic transaminase (GOT) would be present in the blood for a sufficient time period and in sufficient concentration to interact with its substrate, a preliminary pharmacokinetic experiment was conducted. Thus, the pharmacokinetics of GOT were tested over a 48 h period following intra-peritoneal injection. Twenty-four 4-week-old male mice of strain B6SJLF1/J (the background strain of the ALS mutant transgenic mice to be used later in the survival experiment) were purchased from the Jackson Laboratory (Bar Harbor, ME, USA) for the preliminary pharmacokinetic tests. Porcine heart GOT (Lee BioSolutions, Maryland Heights, MO Cat # 300-10-50) was diluted in PBS (5.4 μg/200 μL). Blood samples were taken at various intervals (at minutes 5 and 30, at hours 1, 2, 4, 6, 24, and 48, N = 3–4) following a single IP administration of GOT 2.14 mg/kg based upon previous study using the iv route [15]. Serum was separated by centrifugation and stored at −70 °C pending analysis. The levels of GOT were determined spectrophotometrically using a kit (cat # 23-666-120 Pointe Scientific Canton MI) as prescribed by the manufacturer. Serum concentrations of GOT elevated rapidly and reached an apparent max at 6 h (Figure 1). A decay half-life of ≈18 h was determined using an online resource (Calculator.net at https://www.calculator.net/half-life-calculator.html, accessed on 8 May 2020). Based on these results, a concentration of 2.14 mg/kg GOT was used for the survival experiment.

### 2.3. Survival Experiment

#### 2.3.1. Animals

**Figure 2:**
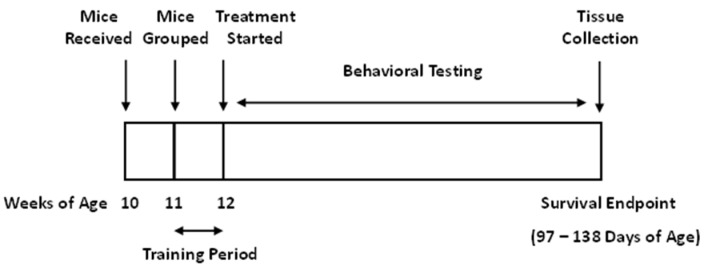
Experimental design for survival study. At week 11, behavioral training was initiated. At week 12, treatments were started.

For experiments testing the effects of treatments on disease progression, 20 male B6SJL-Tg (SOD 1-G93A) transgenic mice (stock number #002726) were purchased from the Jackson Laboratory (Bar Harbor, ME, USA) at ten weeks of age. Since sex can affect the outcome in this model of ALS, only male mice were used. Animals were received at 10 weeks of age and were allowed to adjust to their new environment for a week. They were then distributed into one of four groups such that the mean body weight of each group was optimally uniform, thus controlling for effects due to differences in body weight. See Table 1 for description of treatment groups. Starting at week 12 (the beginning of the experiment), the average weight was 26.4 ± 2.3 g and ranged from 23.4 g to 31.9 g. Mice were singly housed to allow accurate measurements of individual food consumption. All mice were housed in a facility maintained at 21 ± 1 °C with a relative humidity of 55 ± 10% with a 12 h light–dark cycle. From 11 to 12 weeks of age, mice were trained daily on both behavioral research apparatus (accelerating rotarod and hanging wire test) to preclude any learning effect. At 12 weeks of age, treatment and weekly behavioral testing began and continued until mice reached survival endpoint and were euthanized. Figure 2 shows the experimental design.

#### 2.3.2. Treatment Groups and Diets

The supplement complex used as a metabolic therapy is called the Deanna Protocol (DP) and was described previously [20,22]. It consists of four components in combination (each described here as the percentage by weight of the DP-containing diets): 10% L-arginine and alpha-ketoglutarate in a 1:1 ratio (AAKG, Primaforce), 10% medium chain triglyceride oil (MCT oil, Now Sports), and 1% β-phenyl-GABA (beta-phenyl-gamma-aminobutyric acid HCl, Powder City), 0.1% Co-Q10 (coenzyme Q10, Bulk Supplements). The DP was mixed with standard rodent chow, 20% by weight. The glutamate scavenging protocol (GSP) consisted of intraperitoneal (IP) administration of glutamic-oxaloacetic transaminase GOT (2.14 mg/kg) 3× per week, plus 5% oxaloacetic acid (Ox), which was kindly provided by Terra Biological LLC (San Diego, CA, USA) and was combined with chow mix. Saccharin was included in all diets to encourage consumption. Water was included to create paste-like, palatable texture. See Table 2 for a summary of each diet’s components.

Thus, four groups were used. The control group (C; n = 4) was provided standard chow only (2018, Teklad Global 18% Protein Rodent Diet; Envigo), whose macronutrient composition was 18.6% protein, 6.2% fat, and 44.2% digestible carbohydrate. The DP group (n = 5) received the standard chow mixed with the DP mixture. The GPS group (n = 5) received the standard chow plus IP injections of GOT 3X/wk (2.14 mg/kg) and Ox (5% by weight) mixed into the food. The DP+GPS group (n = 5) received the standard chow with the DP and Ox (5% by weight) mixed into the food, plus IP injections of GOT 3X/wk (2.14 mg/kg). Three times per week, food was mixed freshly, weighed, and provided to each individually housed mouse. Food and water were provided ad libitum. All food consumption was measured and recorded. Body weight was monitored weekly. When animals developed substantial motor impairment, a water–gelatin mixture was placed in a standard plastic 100 mm Petri dish on the bottom of the cage to maintain hydration. Since each diet was visually distinct, only the experimenter recording the neurological score was blinded during this study.

#### 2.3.3. Assessment of Motor Function

Motor function was assessed weekly using the accelerating rotarod test and the hanging wire test. One week prior to treatment, mice were familiarized with the equipment and were trained on each test to minimize learning effects during the treatment period. During the treatment period, mice were tested once a week on each task, with at least three days between each test to minimize fatigue. Performance was measured from 12 weeks of age until failure to perform.

Endurance and coordination were measured using the accelerating rotarod test. Mice were placed on a rotarod (Rotamex 5, Columbus Instruments) which accelerated from 0 RPM to 40 RPM over 180 s. The time maintained on the rotarod by each mouse was then recorded. Three attempts were given to each mouse on each testing day. Mice were rested for at least 3 min between trials. The maximum duration achieved by each mouse was used for data analysis.

Neuromuscular function and strength were measured using the hanging wire test. Mice were placed on a mouse cage wire grid in a natural, supine position. The grid was then gently shaken to prompt the mouse to grab on securely. The grid was then quickly and smoothly inverted and rested upon an empty housing box, and the time required for the mouse to release all four paws and drop from the grid was recorded. Each mouse was given three attempts to reach a maximum time of 90 s. Mice were rested for at least 3 min between trials. The maximum duration achieved by each mouse was used for data analysis.

#### 2.3.4. Assessment of Neurological Function

Neurological function was assessed using neurological score based on the criteria described by Ari et al. 2014 (Table 3) twice a week until 109 days of age, and 3–4 times a week thereafter to account for rapid advancement in disease progression. Mice that met survival endpoint were euthanized and recorded as “4” for analysis on subsequent days. A blinded investigator assessed neurological function throughout this study, and the environment was kept consistent to minimize any potential stimuli that could affect score (i.e., changes in lighting or loud noises).

#### 2.3.5. Survival Endpoint

Survival endpoint criteria were defined as meeting one of the following conditions: unable to access food or water, unable to return to the normal upright position within 10 s following push over, or paralysis of both hind limbs. When the mice reached the endpoint, body weight was recorded, and mice were humanely euthanized by CO_2_ inhalation. Organs (brain, heart, lungs, liver, kidneys, spleen, and gastrocnemius and soleus muscles) were harvested immediately for further analysis, and their weights were recorded. This study was conducted with approval of the University of South Florida office of Research Integrity and Compliance protocol number: IS00000655.

### 2.4. Statistical Analysis

For pharmacokinetic analysis, values for serum GOT activity measured at each time point were averaged and plotted. A decay half-life was determined using an online resource (Calculator.net at https://www.calculator.net/half-life-calculator.html, accessed on 8 May 2020). For body weight and organ weight analyses and food consumption analysis, values in intervention groups were compared to control group with Student’s *T*-test. For the accelerating rotarod and hanging wire tests, data were analyzed using GraphPad PRISM version 9.0.1. Significance between groups at each time point was determined by one-way ANOVA with Tukey’s multiple comparison test. Mice that were unable to begin a behavioral test on a given day, either due to weakness or having succumbed to illness, were scored with a time of zero for that day’s test. All data are presented as the mean ± standard error of the mean (SEM). Results were considered significant when *p* ≤ 0.05. For the neurological score, mice that were euthanized are included in the category “4” on the subsequent days for analysis. Neurological score was analyzed using two-way ANOVA repeated measures with Tukey’s multiple comparisons post hoc test. Results were considered significant when *p* < 0.05. Survival was analyzed using the Kaplan–Meier estimator, and correlation curves were plotted with GraphPad Prism Version 9.0.1. Results were considered significant when R^2^ > 0.25.

## 3. Results

### 3.1. Effect of Treatment on Body and Organ Weight

The DP-treated groups showed reductions in body weight compared to the control group.

**Figure 3:**
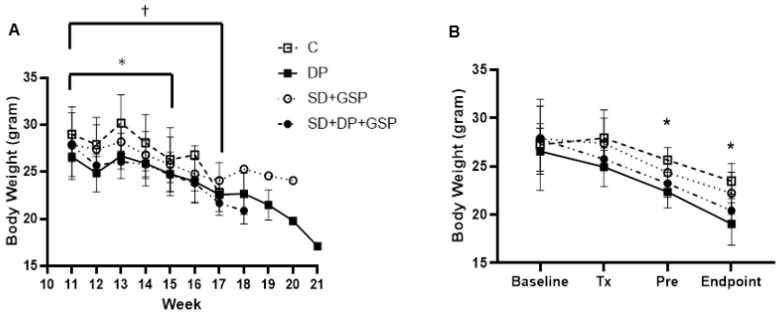
Effect of treatment on body weight. Body weights were measured at weekly intervals—baseline (week 11, when the mice were assigned to treatment groups), Tx (week 12, when treatment began), and every week thereafter until the endpoint. Data points indicate mean +/− SEM. (**A**) Weight change within groups. The body weight of all groups progressively decreased throughout the course of the experiment. A statistically significant reduction from baseline in the weight of each group (*p* < 0.05) was noticed by week 15 for group DP+GSP (*), and by week 17 for the three other groups (†). (**B**) Weight change between groups. The body weights of DP and DP+GSP were significantly less than C at Pre and Endpoint, * *p* < 0.05. Abbreviations: Tx, Beginning of treatment; Pre, 1 week prior to endpoint.

Body weights were measured at baseline (week 11), when treatment began (week 12), every week thereafter, and at the endpoint. The baseline weights (in grams) of the groups were as follows: C = 27.2 ± 4.7; DP = 26.6 ± 2.4; and GSP = 27.9 ± 3.4 and DP+GSP = 27.8 ± 1.6. See Figure 3, B. Starting at week 12, all mice included, the average weight was 26.4 ± 2.3, and ranged from 23.4 to 31.9. Figure 3 shows the effect of treatment on body weight, also shown in Table 4.

In all groups, the body weight progressively declined over time. Within groups, a statistically significant reduction (*p* < 0.05) in the weights of each group, compared to baseline, was noticed by week 15 for group GSP and by week 17 for the other three groups († symbol in Figure 3). At the endpoint of the experiment, animals were weighed, humanely euthanized, and tissues were harvested, weighed, and preserved for subsequent analysis. Between groups, compared to the control group, both groups that received the DP showed a reduction in body weight at the endpoint. The average body weights at the endpoint were as follows for each of these groups: C = 23.5 ± 0.92; DP = 19.0 ± 0.98; GSP = 22.2 ± 0.97; and DP+GSP = 20.4 ± 1.74. See Table 4. Further, compared to the C group, the DP group showed a decrease in the weights of the liver and lower leg muscles. See Table 4.

### 3.2. Effect of Treatment on Food Consumption

The DP-treated groups consumed more calories per day than the control group.

**Figure 4:**
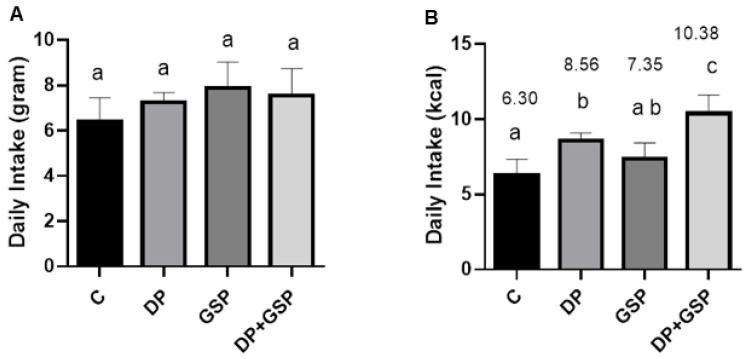
Average daily food consumption. Food consumption was measured every other day and compared between groups. Data bars with shared alphabetic symbols are not significantly different. Data are presented as mean +/− SEM. (**A**) There was no difference in the mass of food consumed. (**B**) There were significant differences between the groups in the energy consumed. Note that the DP+GSP group consumed significantly more daily calories than any of the other groups. Note that the DP group consumed significantly more calories than the C group.

**Figure 5:**
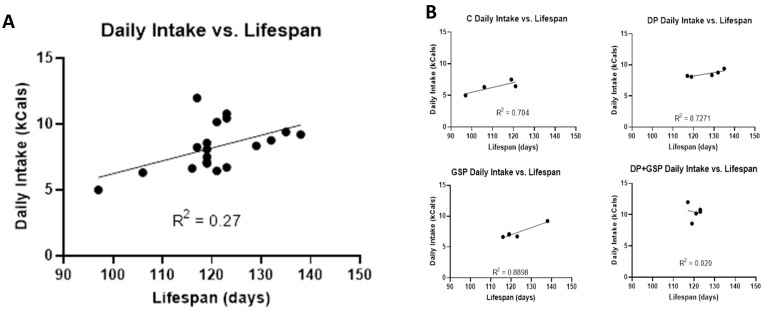
Daily Intake vs. Lifespan. Graphs depicting linear regression of average daily caloric intake versus lifespan. (**A**) When data from all the groups were plotted, there was a positive correlation between average daily food energy consumed and survival (R^2^ = 0.2565). (**B**) When data from each group were plotted individually, three of the groups displayed strong positive correlations (R^2^ values for C, DP, and GSP were 0.704, 0.7271, and 0.8898, respectively). However, no such correlation was found for the DP+GSP group (R^2^ = 0.0203).

There was no significant difference between groups in the weight of food consumed. However, there were significant differences between groups in the calorie intake of food consumed (Figure 4). The average calories consumed per day by each group were as follows: C = 6.30 ± 1.02, DP = 8.56 ± 0.52, GSP = 7.35 ± 1.07, and DP+GSP = 10.38 ± 1.23. The composition of the diets made it impossible to formulate them in such a way as to be equally calorie-dense (Table 2. When the data are plotted to compare the total amount of energy consumed versus lifespan, there is a positive correlation between the two factors (R squared = 0.2686)) (Figure 5).

### 3.3. Effect of Treatment on Motor Function

The DP and GSP groups all showed improvement in motor function.

**Figure 6:**
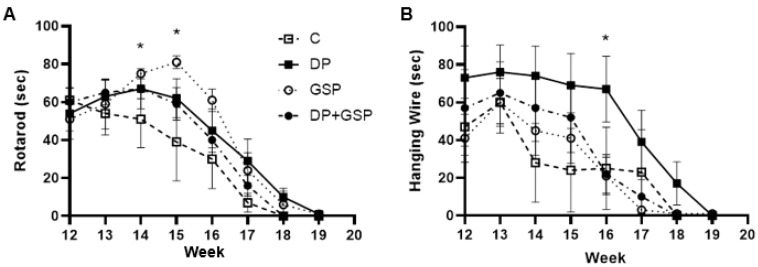
Effect of treatment on motor function. Graphs depicting average time for each group for each week are shown. Data points indicate mean +/− SEM. After one week of training and familiarization with the accelerating rotarod and the hanging wire test, mice were tested once per week on each apparatus. (**A**) Rotarod test for endurance and coordination. Compared to baseline, C mice showed a steady decline in performance beginning at week 13, reaching significance at week 17 (*p* = 0.001). DP mice showed slight statistically insignificant improvement at week 14 before decline began, which reached significance at week 18 (*p* = 0.004). GSP mice showed a significant improvement at weeks 14 and 15 before decline began, reaching significance at week 18 (*p* = 0.006). GSP mice performed significantly longer than C at week 14 and 15, indicated by asterisk (*p* < 0.05). DP+GSP mice remained relatively level up to week 15 before decline began, which reached significance at week 16 (*p* < 0.05). (**B**) Hanging wire test for strength. Compared to baseline, C mice showed a decreased performance by week 14. DP mice remained consistent until week 17 and were significantly higher at week 16 than C (*p* = 0.045). DP mice performed significantly longer than C at week 16, indicated by symbol (*p* < 0.05). GSP mice remained relatively stable up until week 16. DP+GSP mice remained relatively stable up until week 16.

On the rotarod test, compared to baseline, the control group showed a steady decline in performance beginning at week 13, reaching significance at week 17 (*p* = 0.001) (Figure 6A). The DP mice showed slight, statistically insignificant improvement at week 14 before their decline began at week 15, which reached significance at week 18 (*p* = 0.004). GSP mice showed a significant improvement at week 14 (*p* < 0.05) and nearly significant improvement at week 15 (*p* = 0.05) before their decline began at week 16, reaching significance at week 18 (*p* = 0.006). The DP+GSP group maintained performance up to week 14 before their decline began at week 15, which reached significance at week 16 (*p* < 0.05).

On the hanging wire test, control mice showed a decreased performance by week 14 compared to the baseline (Figure 6B). The DP group remained consistent until week 17 and was significantly higher at week 16 than C (*p* = 0.045). The GSP and DP+GSP groups mice maintained stable performance until week 16, followed by a rapid decline.

### 3.4. Effect of Treatment on Neurological Function

The DP and GSP groups all showed improvements in neurological function.

**Figure 7:**
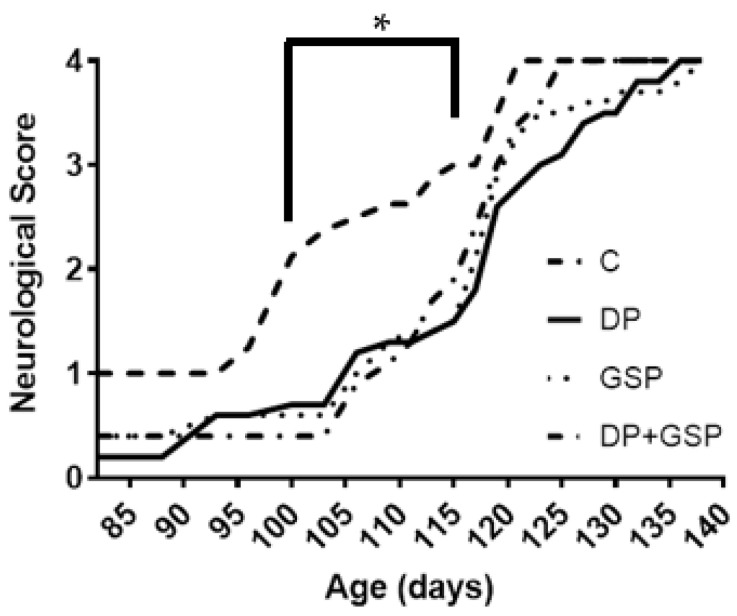
Effect of treatment on neurological deficit. Graph depicting progression of average neurological score over time. DP, GSP, and DP+GSP attenuate neurological deficit in SOD1-G93A. Note that there was no significant difference between treatment groups at baseline (82 days of age) up until 100 days of age. DP mice exhibited significantly lower neurological scores between days 100 and 115 (indicated by asterisk), SD+GSP mice between days 100–109 and days 113–115 (a total of 13 days), and DP+GSP mice between days 100 and 111 (a total of 12 days) (*p* < 0.05). DP thus attenuated neurological deficit the longest, followed by GSP and DP+GSP, respectively. All data are represented by means.

For the sake of clarity, it is important to note that the higher the neurological score is, the greater the neurological deficit. From baseline (82 days of age) up until 99 days of age, there were no significant differences in neurological scores between treatment groups (Figure 7). However, compared to the C mice, the DP mice exhibited significantly lower neurological scores between days 100 and 115 (16 days). Likewise, the GSP mice had lower neurological scores between days 100–109 and 113–115 (13 days), and DP+GSP mice had lower neurological scores between days 100 and 111 (12 days) (*p* < 0.05). DP thus attenuated neurological deficit the longest, followed in order by GSP and DP+GSP.

### 3.5. Effect of Treatment on Survival

The DP and GSP groups all showed a trend for longer survival.

**Figure 8:**
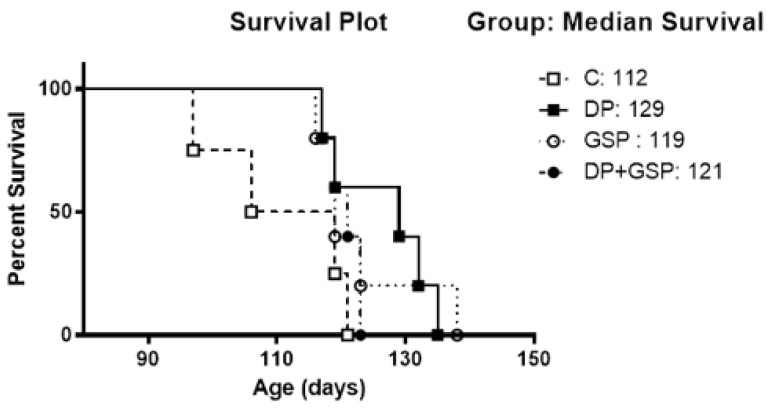
Effect of treatment on survival. A Kaplan–Meier survival plot of study groups shows that the DP group shows a trend toward increasing lifespan. No statistically significant differences (*p* = 0.07). Median survival times (days alive) for each group are also shown.

Animals receiving DP tended to survive the longest compared to control animals (Figure 8). However, this did not reach statistical significance (*p* =.07), likely due to the low subject number. The median survival (in lifespan) of each of the groups was as follows: C = 112, DP = 129, GSP = 119, and DP+GSP = 121. In terms of the percentage increase in lifespan over the control group, these numbers translate as follows: DP = 15%; GSP = 6%; DP+GSP = 8%. The median survival (in days of the experiment) of each group was as follows: C = 20, DP = 47, GSP = 27, and DP+GSP = 29. In terms of percentage increases over the control group, these numbers translate as follows: DP = 135%; GSP = 35%; and DP+GSP = 45%. On day 20 of the experiment, half of the control group had reached the end of life, while all the animals in the other groups were still alive.

## 4. Discussion

The purpose of this study was to compare the previously studied DP [20,21] with the glutamate scavenging approach [18]. In addition, based on our anticipation that both individual treatments would be beneficial, we wanted to investigate the efficacy of a combination of the two therapies. Here, we have presented our findings on the effects of three different treatments on the progression of disease in the SOD-1 G93A mouse model of ALS. The treatment group that received the Deanna Protocol (DP) replicated some of the findings from the previous study on its therapeutic efficacy on motor function, neurological function, and survival [20]. The treatment group that received the Glutamate Scavenging Protocol (GSP) showed significantly improved motor and neurological function and trended toward improvements in survival. The combination of the DP+GSP improved neurological function and tended to extend survival; however, it did not improve motor function. In this regard, the combination of DP and GSP did prove more beneficial than the control group, but not as beneficial as the individual treatments of DP or GSP.

The limitations of this study include the use of the SOD1-G93A mouse model, which does not address sporadic ALS or other genetic variants of ALS, such as the TDP-43 and the FUS/TLS gene mutations. The SOD1-G93A transgenic mice carry mutations that replicate early onset ALS, and these represent approximately 10% of ALS cases, suggesting that a genetic model is likely not representative of the majority of ALS cases. The major weakness of this study is the low sample number. Assumptions concerning the validity of a model of disease are important to address. Each of the animal models currently available to study ALS has advantages, as discussed in detail elsewhere [7]. On the pro side, the SOD1-G93A is the most widely used, genetically based model currently available. It faithfully reproduces many of the aspects of the human ALS condition, including progressive motor dysfunction, loss of motor neurons, glutamate toxicity, and mitochondrial dysfunction, and it contains a multiple copy transgene coding for the mtSOD1 variant most common in ALS patients. These effects manifest systematically and are described by resources made available by The Jackson Laboratory, which describe the phenotype, in part, as showing decreased grip strength, impaired coordination, motor neuron degeneration, severe muscle weakness beyond 3 months old, hind limb tremors at 14 weeks old, paralysis in one or more limbs due to loss of motor neurons in the spinal cord, and abbreviated lifespan. On the con side, the early and unpredictable onset of symptoms in this model makes the timing of interventions problematic.

The findings in our present study correlate well with our previous DP study and are also comparable with other studies described in the literature. In our previous study, the symptoms had already appeared on the first day of tracking the neurological score, which was on day 100. Thus, for the present study, we started tracking the neurological score earlier in life. Still, symptoms had already appeared at the start of tracking the neurological score at day 82. In the earlier study, during motor function testing, a decline in performance began around day 105. Similarly, in the current study, the decline began around day 105. In the previous study, the mean lifespan in the control group was 120 days, and 129 days in the DP group. In the present study, the mean lifespan in the control group was 112 days, and 129 days in the DP group.

The outcome measures used for this mouse study were those deemed relevant to the pathognomonic correlates in humans. Body weight change was tracked, as loss of weight is a cardinal sign of the onset of symptoms in this mouse model, and loss of weight is an important prognostic indicator for survival in ALS patients [24]. During the training period between week 11 and week 12, the weights of all the mice began to slowly decline. Thus, the treatments were initiated (week 12) after the disease had manifested. Timing of interventions is important when considering the impact of translational medicine since medical treatment for ALS patients can only reasonably be considered after symptoms manifest. Food consumption was closely monitored, as in ALS patients, malnutrition is highly correlated with poor outcomes [25]. As motor function declines with the progression of ALS, the changes in rotarod performance were used to determine the effect of disease and treatment on coordination. The hanging wire test was used to measure changes in muscle strength. The neurological score was designed to assess hind limb function since that is the earliest neurological sign to emerge in the SOD1-G93S mice [20], and it allows for unbiased quantification of disease progression. While the prior measures are all related to quality of life, the final measure, survival, is important to quantify the potential effects of treatments on the length of lifespan.

Each component in the DP is used for a rational, physiologically based reason. First, multiple studies suggest that ALS can be viewed as a neurovascular disorder with vascular abnormalities preceding neurological damage [26]. AAKG is composed of equal parts arginine and alpha-ketoglutarate. Thus, our treatment provided about 5% arginine by weight of the diet. Interestingly, a previous study showed that 6% L-arginine in drinking water preserved spinal neurons and improved outcomes in SOD1 mice [27]. The role of arginine in endothelial relaxation has been known for decades [28]. However, while arginine supplementation has not been shown to reliably improve blood flow in healthy individuals, it has been shown to be useful in treating various disorders associated with vascular disease [29,30]. Thus, arginine may provide a benefit in diseased states either by directly enhancing blood flow and/or as a substrate in creatine synthesis [31].

Second, ALS is also associated with several defects in energy metabolism, glucose utilization, mitochondria function, and insulin resistance [32]. In ALS patients, progressive muscle weakness leads to dysphagia and lowered food intake, and weight loss correlates with poor clinical outcomes [25]. Therefore, alternate forms of energy may be expected to provide some measure of relief. Indeed, a high-fat diet was shown to extend survival in the SOD1 mice [33]. To address energy and metabolism defects, the DP provides alternative sources of energy, including alpha-ketoglutarate, which can enter the TCA cycle independent of insulin signaling, and MCT oil, which can enter the liver directly and be utilized rapidly for ketone production [34]. It was also described that the progression of mitochondrial chain dysfunction correlates with the advancement of disease [35]. The purpose of including Co-Q10 in DP is to provide support to the respiratory chain of the mitochondria. However, previous studies using the oxidized form of Co-Q10 produced slightly positive results in mice [36] and no benefit in humans [37]. The use of a stabilized form of Co-Q10 was ineffective in mice [38]. While increasing evidence suggests that the DP does help in this model, it is not clear whether the Co-Q10 used in the DP actually participated in the apparent benefits. Further studies are necessary to determine the contributing effect of each component of DP. In ALS, spasticity and fasciculations originate from both central and distal effectors [39]. As a blood–brain barrier-permeable GABA analog, the purpose of β-phenyl-GABA is as a generalized anti-convulsant, anti-spastic agent [40].

Additionally, Numerous neurological pathologies, including stroke, traumatic brain injury, Parkinson’s disease, and ALS, have been associated with glutamate excitotoxicity [41]. Blood glutamate scavenging has been successful in numerous preclinical studies [16,17,18,42,43] using either GOT or Ox alone, or a combination thereof. In our study, the GOT enzyme was injected intraperitoneally and produced blood concentration effects similar to that seen with intravenous administration in other studies [15]. The Ox was mixed with food and consumed ad libitum. The combination of the GOT and Ox apparently improved the rate of decline of symptoms in the GSP treatment group.

Thus, both the DP and the GSP are combination therapies with a clear theoretical purpose for each individual component [44]. Each of these therapies showed improvements in the model used in this study. However, the combination of these approaches did not result in an additive effect. Indeed, the major effect of the combination of DP and GSP was an increase in the number of calories consumed. It is unclear what mechanisms might underlie these apparently contradictory effects. However, consider the following: a major component of the DP is alpha-ketoglutarate, and its purpose is to provide energy by entering the TCA cycle directly. The major purpose of the GSP is to provide an enzyme (GOT) and substrate (Ox) to remove peripheral glutamate in a reaction that produces more alpha-ketoglutarate. However, this enzyme is bi-directional and substrate-dependent; therefore, it is possible that the exogenous supplementation of the alpha-ketoglutarate may restrict or reverse the intended effect of the GOT-mediated reaction.

Another potential confound is the effect of exogenous substances on the TCA cycle. For instance, the idea behind using the Ox is that when working synergistically with GOT, it promotes the glutamate scavenging process. Likewise, the putative purpose of the AAKG is to act as a distinct energy source by providing AKG to the TCA cycle directly. However, the Ox can also be utilized as a TCA cycle intermediate. The entry point into the TCA cycle for each of these substrates is basically opposite each other. If the effects of mass action were to prevail, one might expect these components to be mutually antagonistic in the TCA cycle. Thus, whatever benefits are derived from the action of the GOT/Ox might be negated when the AAKG is added to the mix, and vice versa. However, if the action of the GOT is rapid enough in the blood, then the primary effect of the GOT/Ox would indeed be that of a glutamate sink. In fact, it has been shown that oxaloacetate is able to cross the blood–brain barrier in mice [45,46], while any large enzyme such as GOT is not. Therefore, it is also possible that the Ox enters the central nervous system and is not available to interact with GOT. It has also been shown that AKG functions as an anti-oxidant reacting with hydrogen peroxide [47]. It also can impact gluconeogenesis via its conversion into glutamate, which then can be converted to glutamine [48]. Clearly, the actions of these compounds, both individually and in combination, are complex phenomena. Further, targeted experiments would be needed to elucidate the mechanisms at work.

Another interesting finding in this study concerned the effect of treatments on food consumption and body weight. One feature long associated with ALS patients is loss of body weight and the inability to eat enough to maintain a positive energy balance [49]. A severe loss of appetite has been reported in 50–60% of ALS patients [50]. Indeed, a decrease in body mass highly correlates with poor prognosis in ALS [51,52], while a high-fat, high-energy diet has been shown to help in the SOD1 ALS model [53]. Not surprisingly, in the study presented here, in each group, over the course of treatment, body weights declined as a consequence of disease progression. Body weights were compared over a series of time points, including “Baseline” (when animals were assigned to their groups), “Tx” (when treatment commenced), “Pre” (the week prior to the animal’s termination), and at “Endpoint” (Figure 3B). Both the DP and the DP+GSP groups weighed significantly less than the control group at the Pre and Endpoint time points. This was even though these two groups also consumed more food energy per day than the SD group. Moreover, both DP groups had the longest median survival times. It is tempting to speculate that the DP prolongs life by promoting increased consumption, which allows for positive energy balance and prolonged life, despite the reduction in body weight, but additional studies are required to delineate such mechanisms. Furthermore, the DP group showed a significant reduction (*p* < 0.05) in the weight of the liver and muscle, unlike in our previous study.

Another unusual finding concerned the relationship between food energy intake and survival. When data from all the groups were plotted, there was a positive correlation between average daily food energy consumed and survival (R^2^ = 0.2565). When data from each group were plotted individually, three of the groups displayed strong positive correlations (R^2^ values for C, DP, and GSP were 0.70, 0.73, and 0.89, respectively). However, no such correlation was found for the DP+GSP group (R^2^ = 0.020). This result supports the hypothesis that the DP and the GSP approaches are fundamentally oppositional in outcome, and perhaps in mechanism as well. It is interesting to note that the most positive behavioral and survival outcomes were observed in the group that lost the most weight. The DP group showed improvements in all major outcomes and consumed more than the control group, yet weighed the least at the end of life.

For the most part, for each individual mouse, food consumption did not change over time, with animals eating consistent amounts up until symptoms interfered with eating, which coincided with weight loss 2–3 days before the end of life. Therefore, we should not construe the loss of weight per se to be causative for slowing the decline in performance observed in the DP group. However, rather, the loss of weight more likely reflects the effect of the treatment on the preservation of the animals’ health, which allowed them to survive longer despite the progressive loss in weight. There was no correlation between baseline weight and lifespan, baseline weight and daily caloric intake, endpoint weight and lifespan, or endpoint weight and daily caloric intake.

The present study was not designed to elucidate detailed mechanisms. In general, we had theorized that the DP should work by repairing defective cellular energetics downstream of the SOD-1 mutation. While SOD-1 is found in all tissues and cell types, and the hallmark feature of ALS is the failure of neurons [54], it has been shown that the cells that initiate the cascade of events that underlies ALS etiology are most likely the glial cells [55,56,57] and muscle cells [58,59]. Glial cells are the major repository of glutamate in the CNS, and when properly functioning, they maintain extracellular levels of glutamate at safe concentrations. When the integrity of glial cells is compromised, glutamate can leak from the cells and increase in the extracellular space leading to glutamate excitotoxicity.

Combination therapies are increasingly becoming the focus of researchers. Recent work has demonstrated the efficacy of combining components of the DP and GSP in rescuing damaged neurons [22]. Human motor neurons derived from ALS patients display axonal pathology remarkably similar to that seen in human motor neurons exposed to toxic levels of glutamate. In each situation, damaged neurons were rescued by the administration of a combination of AAKG, GABA, serotonin, and GOT. When either of those components was omitted, the treatment failed. Thus, it is unclear why, in the present mouse study, the combination of these two approaches was inferior to either approach alone. It is unclear whether, in the whole animal model, the DP is affecting neurons or glial cells, or both. Future research should focus on understanding the role of astroglial cells in this process, perhaps by employing mixed cell-culture systems.

## 5. Conclusions

ALS is a divergent syndrome with unclear mechanisms of onset, with potential causality from genetic and environmental factors. In general, many agents identified as therapeutic in animal models have not proven to be beneficial in humans. Specifically, there has long been a concern in the ALS research community regarding the validity of SOD1 animals as translational models for the human condition [60]. Therefore, in our lab’s previous study, we took a reverse approach. Since many ALS patients had already used the DP (which consists of safe, over-the-counter supplements) and found it to be helpful, our lab investigated and was able to demonstrate its benefit in the SOD1-G93A mouse model as well [20]. We have now extended these findings and confirmed measurable benefits in motor function, neurological outcome, and a trend in extended survival with the DP. The glutamate scavenging approach also showed clear and measurable improvements in this mouse model, in both motor function and neurological outcome, similar to what others found using a rat model of ALS [18]. Because ALS is not a discreet disease, different patients will likely benefit from different forms of treatment. Considering these results, however, implementing either a DP or GSP approach merits an investigation in clinical trials.

## Figures and Tables

**Table 1:** Experimental treatment groups.

Treatment	Group Name	Sample Size
Control	C	4
Deanna Protocol	DP	5
Glutamate Scavenging Protocol	GSP	5
Deanna Protocol + Glutamate Scavenging Protocol	DP+GSP	5

**Table 2:** Diet composition. Percentages of each dietary component by weight are shown. The caloric density of C, DP, GSP, and DP+GSP diet preparations were 1.67, 2.60, 1.58, and 2.47 kcal/g., respectively. Abbreviations: Std., standard; AAKG, arginine-alpha-ketoglutarate; MCT, medium-chain triglyceride oil; Co-Q10, coenzyme Q_10_.

Component	C	DP	GSP	DP+GSP
Std. Chow Mash	53.8	42.4	51.1	40.3
Water	46.1	36.1	43.8	34.3
Saccharin	<0.1	<0.1	<0.1	<0.1
AAKG	0	10.2	0	9.7
MCT	0	10.2	0	9.7
β-phenyl-GABA	0	1.0	0	1.0
Co-Q10	0	0.1	0	0.1
Oxaloacetic Acid	0	0	5.0	5.0

**Table 3:** Criteria of the neurological score. The system used to quantify the level of neurological decline is presented.

Score	Description of Movement Behavior
0	Hind legs are fully extended during tail elevation
1	Hind legs are not fully extended during tail elevation
1.5	Abnormal gait
2	Toes curl under while walking
2.5	Difficulty walking but still using all four legs
3	Dragging hind legs while walking, partial leg paralysis
3.5	Single hind leg paralysis
4	Complete hind limb paralysis or unable to return to upright position within 10 s

**Table 4:** Body and organ weights. The weights of body and harvested tissues following necropsy and dissection are presented. Data are presented as mean +/− SEM. Rows in bold indicate components in which weights significantly differed from the C group. * *p* < 0.05, ** *p* < 0.01.

Weights	C	DP	GSP	DP+GSP
Body (g)	24 +/− 0.92	19 +/− 0.98 **	22 +/− 0.97	20 +/− 1.74 *
Brain (mg)	443 +/− 23.00	388 +/− 14.85	398 +/− 10.13	386 +/− 14.49
Heart	138 +/− 10.90	130 +/− 13.61	142 +/− 4.36	137 +/− 5.97
Lungs	280 +/− 6.33	197 +/− 26.18	257 +/− 40.68	196 +/− 14.15
Liver	1084 +/− 70.61	639 +/− 224.61 *	1138 +/− 21.14	1034 +/− 38.53
Kidneys	366 +/− 110.55	388 +/− 19.68	495 +/− 32.7	534 +/− 17.28
Spleen	93 +/− 20.43	70 +/− 8.21	68 +/− 7.94	65 +/− 7.66
Muscle	111 +/− 8.74	63 +/− 12.66 *	90 +/− 14.25	84 +/− 7.05

## Data Availability

The raw data used for this study will be made available by the corresonding author as resonably requested.
